# Characterization of Diverse Internal Binding Specificities of PDZ Domains by Yeast Two-Hybrid Screening of a Special Peptide Library

**DOI:** 10.1371/journal.pone.0088286

**Published:** 2014-02-04

**Authors:** Yi Mu, Pengfei Cai, Siqi Hu, Sucan Ma, Youhe Gao

**Affiliations:** 1 National Key Laboratory of Medical Molecular Biology, Department of Physiology and Pathophysiology, Institute of Basic Medical Sciences, Chinese Academy of Medical Sciences and School of Basic Medicine, Peking Union Medical College, Beijing, P.R. China; 2 MOH Key Laboratory of Systems Biology of Pathogens, Institute of Pathogen Biology, Chinese Academy of Medical Sciences and Peking Union Medical College, Beijing, P.R. China; Harvard Medical School, United States of America

## Abstract

Protein-protein interactions (PPIs) are essential events to play important roles in a series of biological processes. There are probably more ways of PPIs than we currently realized. Structural and functional investigations of weak PPIs have lagged behind those of strong PPIs due to technical difficulties. Weak PPIs are often short-lived, which may result in more dynamic signals with important biological roles within and/or between cells. For example, the characteristics of PSD-95/Dlg/ZO-1 (PDZ) domain binding to internal sequences, which are primarily weak interactions, have not yet been systematically explored. In the present study, we constructed a nearly random octapeptide yeast two-hybrid library. A total of 24 PDZ domains were used as baits for screening the library. Fourteen of these domains were able to bind internal PDZ-domain binding motifs (PBMs), and PBMs screened for nine PDZ domains exhibited strong preferences. Among 11 PDZ domains that have not been reported their internal PBM binding ability, six were confirmed to bind internal PBMs. The first PDZ domain of LNX2, which has not been reported to bind C-terminal PBMs, was found to bind internal PBMs. These results suggest that the internal PBMs binding ability of PDZ domains may have been underestimated. The data provided diverse internal binding properties for several PDZ domains that may help identify their novel binding partners.

## Introduction

The human genome encodes 20,000∼25,000 protein-coding genes, which is much lower than previous estimates (∼100,000 in 2001) [1]. The number was only is approximately 30% more than that of the roundworm *Caenorhabditis elegans* (∼18,000), which once led to the speculation that alternative splicing may occur more frequently in humans than invertebrates. Further investigation revealed no major differences in the frequency of alternatively spliced genes among humans and any of the other animals tested, including *C. elegans* [2]. Thus, the phenomenon may denote the presence of more diverse modes of protein-protein interactions (PPIs) than currently known to exert complex physiological functions in higher organisms. Although a lot of information on PPIs has been released publicly on the benefit of advances in proteomics technologies [3], the identification of promiscuous weak or transient protein interactions, which may play critical roles in cellular physiology, remains a challenge [4].

Protein domains, such as SH2 (Src Homology 2), SH3 (Src Homology 3), PDZ (PSD-95/Dlg/ZO-1), and WW domains, are abundant PPI modules that mediate a variety of biological and cellular functions [5]–[9]. These domains have different recognition motifs for their target ligands; for example, SH2 can recognize tyrosine-phosphorylated (pTyr) motifs, SH3 and WW can recognize proline-rich sequences containing PxxP or PPxY motifs, in which x denotes any amino acid [10], and PDZ domains can usually bind to 4∼5 C-terminal amino acids of target proteins [11]. Besides, non-canonical interaction modes have also been reported [12]–[16], which could contribute to the diversity and complexity of PPIs. And our understanding of non-canonical modes of interaction can be facilitated by systematically exploring the binding properties of those domains.

Most PDZ domains are involved in the recognition of the extreme C-terminal PDZ-domain binding motifs (PBMs) of target ligands; however, accumulating evidences have revealed that some of them can also bind internal PBMs of partner proteins (Table 1). Efforts have been made to develop safe and effective drugs to treat diseases by targeting the internal binding sequences of PDZ domains [17], [18]. However, as limited information about the internal ligand binding specificities of PDZ domains is available, more efforts are needed to systematically explore this non-canonical mode of interaction. Usually, conventional cDNA libraries full of high-abundance and diverse C-terminal ligands were screened against the PDZ domains, which might be unfavorable for the discovery of the internal binding pattern. Here, we speculate that more internal recognition patterns might be found once the diverse C-terminal motifs in the system are ruled out.

**Table 1:** PDZ domains reported to bind internal PBMs.

Protein	PDZ No.	Partner	Internal sequences	References
INAD	5	NORPA	AA972-1070	[19]
PTP-BL	2	RIL	AA249-309	[20]
PTP-BL	4	RIL	AA249-330	[20]
Syntrophin	1	nNOS	-LETTF-	[14]
PSD95	2	nNOS	-LETTF-	[21]
PSD95	1	Kv1.5	AA92-149	[22]
NOS1	1	Vac14	-GDHLD-	[23]
Par6B	1	Sdt/Pals1	-HREMAV-	[24]
Dvl1	1	Frizzled 7	-KTXXXW-	[25]
Dvl1	1	Idax	-KTXXXI-	[26]
SCRIB	4	TBEV-NS5	-EMYYS-	[27]
Harmonin	1	CDH23	AA91-184	[28]
NHERF1	2	Megalin	-ETII-	[29]
NHERF2	1	mSRY	AA93-103	[30]
GRASP55	1	GRASP55	-IGYGYL-	[31]
Dvl2	1	Phage*	WKDYGWIDGK, etc.	[18]
HtrA1	1	Phage*	GVTWGEVLGALV, etc.	[32]
HtrA2	1	Phage*	SHWWGGWL, etc.	[33]
HtrA3	1	Phage*	GVVVDEWVLSLL, etc.	[32]
GIP	1	Phage*	ESSVDLLDG, etc.	[34]
GIP	1	DTX1, STAU1	S/T-X-V/L-D	[35]
Deg2	2	Deg2	-YIIVAG-	[36]
Syntenin-1	1	Syntenin-1	ELSQYMGLSL	[37]
GRASP65	1	GRASP65	-IGYGYL-	[38]
SHANK	1	GluR delta2	S segment	[39]
DLG1	1	Phage*	EETDIW, etc.	[40]

* : Based on the N-terminal phage display peptide library screening.

**Figure 1:**
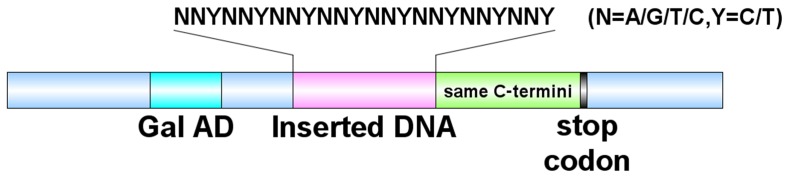
A schematic diagram showing the design of a nearly random internal octapeptide library.

For some PDZ domains, no C-terminal ligands were screened out in the C-terminal phage-display peptide system [41], raising the possibility that these PDZ domains may have a preference for binding internal motifs. The yeast two-hybrid (Y2H) system is another powerful and high-throughput method for studying PPI, which can determine one-to-one interactions, that means when one weak interaction occurs in a single yeast cell, it will not be inhibited by other strong ones. Typically, the main consensus motif for PDZ domains is limited to the three ∼ five residues, so a library with internal octapeptide may cover these core binding motifs. To ensure that the interactions occur between the internal motifs and PDZ domains, all clones in the library should bear an identical C-terminal sequence that is not any type of C-terminal motif for PDZ domains. Based on these criteria, an internal octapeptide library was designed as shown in Figure 1. To avoid pre-termination, the inserted internal DNA fragment was designed as (NNY)_8_, (N = A/G/T/C, Y = C/T), which will not encode any of the three stop codons: TAA, TAG, and TGA. Further, we symmetrically explore the potential internal sequence binding properties of a panel of PDZ domains by screening this special library in the Y2H assays.

## Materials and Methods

### Preparation of bait plasmids

The information of 24 PDZ domains from 18 proteins was shown in Table S1. The boundaries of the PDZ domains were mainly determined in the UniProt database. The human ZO-1 PDZ1-pMBa, PDZ2-pMBa, and PDZ3-pMBa plasmids were kindly provided by Dr. Ben Giepmans (The Netherlands Cancer Institute). For cloning other domains, the gene fragment encoding each domain was respectively amplified by Phusion DNA polymerase (New England Biolabs, NEB) from commercial plasmids purchased from ProteinTech Group and Source BioScience. All sequences of PCR primers were listed in Table S2. The PCR was performed with an initial denaturation for 1 min at 98°C. Ten PCR cycles were performed: 98°C for 5 s, 47°C for 30 s, and 72°C for 5 s, followed by twenty PCR cycles: 98°C for 5 s, 54°C for 30 s, and 72°C for 5 s, and the final extension was 3 min at 72°C. The PCR product was digested with restriction enzymes, and cloned into GAL4 BD vector, pBridge (Clontech Laboratories, Palo Alto, CA). The recombinant plasmids were transformed into DH5α (DE3) *Escherichia coli* and positive clones were selected for DNA sequencing.

### Construction of random octapeptide internal sequence library

An oligonucleotide 5′-TAG GGG *GAATTC* GCT (NNY)_8_ TAC G*GGATCC*AT CGA-3′ (N is an equal mixture of A, G, T, and C; Y is an equal mixture of C and T), encoding 8 random codons in the middle, which flanked by *EcoR* I and *Bam*H I restriction sites, was synthesized to construct a random peptide library. PCR was performed with 240 pmol of the oligonucleotide and 240 pmol of the primer 5′-TCGATGGATCCCGTA-3′ in a total volume of 120 µl. The complementary chain of the template was synthesized by program included denaturation at 94°C for 1 min; followed by annealing at 42°C for 10 min, and extension at 50°C for 10 min. After double digestion with *EcoR* I and *Bam*H I, the products were recovered by short DNA fragment rapid recovery kit and ligated into pGADT7 AD vector, which predigested with the same enzymes. A negative control, in which no insert DNA fragments were added to the ligation reaction, was performed to monitor vector self-ligation. The overnight ligation products were desalted and transformed into electrocompetent cells at 1.8 kv with *E. coli* Pulser Apparatus (BioRad). The transformation product was recovered in 1 mL of LB broth in 37°C for 1 h at 200 rpm. Transformation product (1 µl) was diluted 1∶1000 times. The diluted product was spread on a pre-warmed LB-Amp plate and incubated at 37°C overnight until colonies appeared. The numbers of the colonies were counted and the transformed clones calculated with: Transformed clones  =  (Colony number/Volume plated (µl)) ×dilute times×1000 µl/ml [42]. To evaluate the library, fifty clones were randomly selected for colony PCR with primer pairs (forward primer, 5′-CTATTCGATGATGAAGATACCCCA-3′, and reverse primer, 5′-CTTGCGGGGTTTTTCAGTATCTAC-3′). The plasmids extracted from these clones were further sequenced.

### Y2H screening of the internal peptide library

Each GAL4 BD-fusion bait plasmid was transformed into the yeast strain CG1945 using the lithium acetate protocol. The transformants were grown on SD/-Trp plates and lacZ assays were performed to examine self-activation. The transformants that had no background growth or had background growth but could be inhibited by 3-amino-1,2,4-triazole and were also negative for the LacZ assay were selected for the subsequent screening. The random octapeptide library was screened following the MATCHMAKER Two-Hybrid System protocol (Clontech).Approximate 10^7^ *Trp^+^Leu^+^* transformants were selected on plates with SD -Trp-Leu-His medium in the primary screening and then tested by the improved LacZ assay in the second screening. After rescue, the potential positive plasmids were isolated and retransformed into the yeast strain CG1945 containing corresponding bait plasmid. Only the clones that were positive for all the reporter assays and confirmed by at least two independent tests were selected for specific interactions and sequenced [43].

### Analysis of the consensus-binding sequences

Internal PBMs for each positive PDZ domain were aligned with ClustalX1.83, respectively, and further refined by GeneDoc software. Consensus sequences were deduced from sequence alignments.

## Results and Discussion

### Construction and evaluation of the nearly random octapeptide library

**Figure 2:**
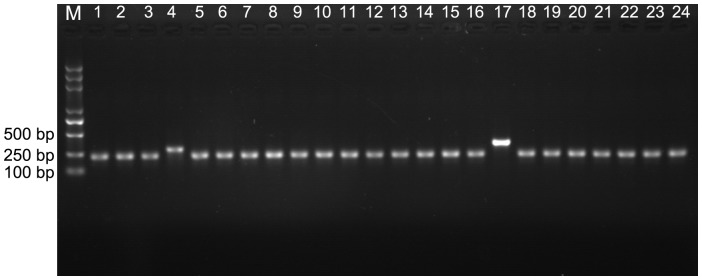
Library quality control by colony PCR. M: DNA ladder 2000 plus; lanes 1–24: PCR product of 24 randomly selected transformants from the internal octapeptide library, respectively.

Y2H screening is usually used as a tool for studying protein-peptide and domain-peptide interactions. Different kinds of libraries were constructed according to the characteristics of the objective peptide ligands. To explore the internal binding properties of PDZ domains in the Y2H system, a random library with highly abundant and diverse internal sequences should be constructed. To ensure the diversity of the library, the complementary chain of the template was directly synthesized, but not via PCR procedure. The double strand cDNA template appeared as a clear 54-bp band on 15% PAGE gel (Figure S1). The ligation and subsequent electrotransfection generated ∼1.0×10^7^ transformed clones. We evaluated the recombination frequency and diversity of our library. Fifty clones were randomly selected from the library and identified by colony PCR for further sequencing. The library may have a negligible number of empty vectors; however, in some clones, the insertion sequence was elongated due to multiple DNA fragments self-ligation (Figure 2). No repetitive insertion sequences were detected among the fifty clones, and their deduced peptide sequences were unique. However, as (NNY)_8_ was designed as the inserted internal sequence, five amino acids M, K, E, Q, and W were excluded from their corresponding peptide sequences.

### Y2H screening of the internal binding sequences of 24 PDZ domains

A total of 24 PDZ domains derived from different proteins that participate in various biological processes and mediate different functions were chosen to screen the octapeptide library (Table S1). Fourteen of the PDZ domains exhibited an ability to bind internal sequences, of which eight were previously reported and six were reported here for the first time. No positive clone was probed against 10 PDZ domains (Table 2). Sequence information for all internal PBMs was shown in Table S3.

**Table 2:** Summary of Y2H screening of internal PBMs against 24 PDZ domains.

No.	PDZ domains	Reported to bind internal PBMs	Confirmed to bind internal PBMs	Consensus
				
1	ZO1-1	+	+	+
2	RIMS2-1	+	+	+
3	GOPC-1		+	+
4	Hyarmonin-1	+	+	+
5	Dvl2-1	+	+	+
6	HtrA2-1	+	+	
7	Par6A-1	+	+	+
8	NHERF1-1	+	+	
9	NHERF1-2	+	+	
10	LNX2-1		+	+
11	Par3L-1		+	
12	Dlg5-3		+	
13	Whirlin-3		+	+
14	Syntenin1-2		+	+
15	CASK-1			
16	PICK1-1	+		
17	Syntenin1-1	+		
18	Scrib-4	+		
19	NHERF2-1	+		
20	NHERF2-2	+		
21	ZO1-2			
22	ZO1-3			
23	DLG5-2			
24	Syntrophin-1	+		
SUM	24	14	14	9

### Binding specificities of internal sequences for each positive PDZ domain

**Figure 3:**
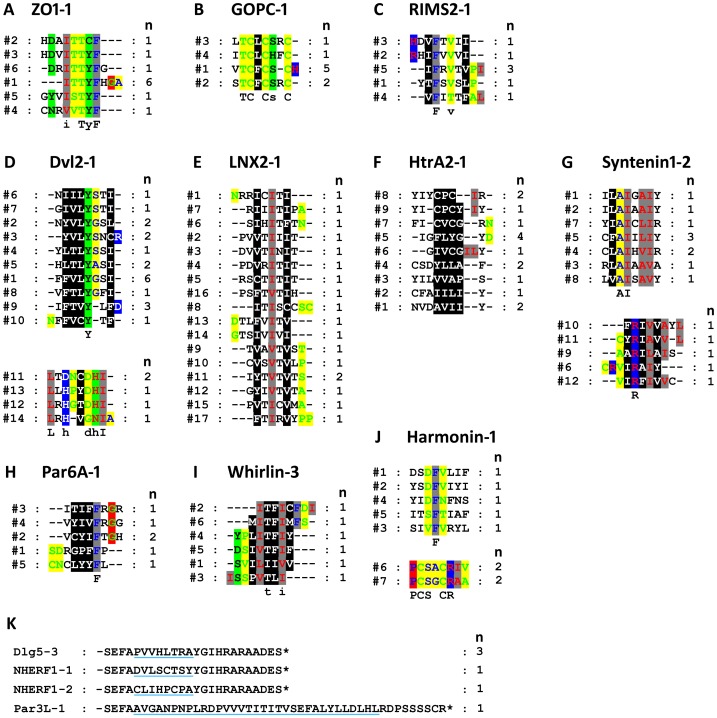
Peptide sequence alignment of internal PBMs screened from the special internal octapeptide library using different PDZ domains as baits. Sequences were aligned by ClustalX 1.83, and further refined by GeneDoc software. A-J) Sequence alignment for the PDZ domain of ZO1-1, GOPC-1, RIMS2-1, DVL2-1, LNX2-1, HtrA2-1, Syntenin1-2, Par6A-1, Whirlin-3, and Harrmonin-1, respectively. Chemical property mode shading was used to highlight sequence residues that share a defined set of properties. K) Internal peptides bind to the PDZ domain of Dlg5-3, NHERF1-1, NHERF1-2, and Par3L-1, respectively. Potential internal PBMs were underlined with blue lines. n, number of clones probed.

(a) ZO1-PDZ1: ZO-1 is a scaffold protein of the tight junction complex that is known to bind α-actinin-4 [44] and the integral tight junction proteins occludin and claudins [45], [46]. The ZO1-1 domain has been suggested to bind the NS5 proteins of dengue virus (DENV) via an internal sequence recognition mechanism [47]. After alignment of the internal PBMs of ZO1-1, a consensus can be deduced as: [**I**/V]-[**T**/S/V]-**T**-[**Y**/C]-**F**, where bold indicates high frequency (Figure 3A). The most important feature of the consensus is that the aromatic amino acid F and polar amino acid T were relatively conserved. The internal sequence binding specificity of ZO1-1 was similar to its core C-terminal PBM obtained from phage peptide screening, Φ-[T/S/R/K]-[S/T]-[Y/W]-[V/I/L]_COOH_ (where Φ is a hydrophobic residue) [41], [48], but some differences were found between them at particular sites. For example, the last position of the internal motif preferred the aromatic amino acid F, but not the aliphatic amino acids V, I, and L. Also, within the internal motif, the polar amino acid T, rather than S, was preferred. The nature of the hydrophobic residue at β2-1 was previously suggested to be a key determinant of the specificity of the last position (P^0^) of the C-terminal motifs of PDZ domains [49]. As ZO1-1 contains an Ile at β2-1, it can likely accommodate the large Phe at P^0^ with a less buried surface. For C-terminal PBMs of ZO1-1, both W and Y were preferred at P^−1^, which was shown to interact with the Asp in position β3-5 [49]. As our system ruled out the aromatic residue W, the amino acid with similar properties, Y, appeared at this position of the internal consensus. Alternatively, ZO1-1 readily accepted another internal peptide, AAAAYIIS (not shown in Figure 3A), which differed from the core consensus and presented as a non-consensus internal ligand for this domain. The N-terminal side of four internal sequences (#2, #3, #4, and #6) contained charged amino acid residues (H, D, R), indicating that they might interact with other charged residues within the loops between β2 and β3, or β4 and α2 of the PDZ domain, similar to the Par-6 PDZ-Pals internal ligand interaction [24].

(b) GOPC-PDZ: GOPC has been reported as a canonical class I PDZ protein that can bind to SSTR5 (-QT**S**[K/R][**I/L**]*), cadherin 23 (-I**T**EL*), hFrizzled 8 (-L**S**QV*), and neuroligin (-ST**T**RV*) (* represents the stop codon of the peptide) via its PDZ domain [50]–[52]. In the present study, GOPC-PDZ exhibited the ability to interact with internal sequences, which can be described as an apparent consensus: [VILS]-**T**-**C**-[FL]-**C**-[**S**H]-R-**C**- (Figure 3B). Similarly, within the consensus, an analogous class I PBM, **T**-**C**-[FL], was observed. The amino acid composition was relatively conservative at several positions, such as T other than S, and L or F, but not other hydrophobic amino acids, were respectively preferred at the second and fourth positions of the internal consensus. In addition, residue C appeared frequently, interspersed in the internal sequences. At P^−1^, the relatively large amino acids K, R, E, and Q were selected in canonical C-terminal ligands of GOPC-PDZ, whereas the small amino acid C was strictly preferred at the corresponding site of the putative internal binding motif **T**-**C**-[FL]. Similar to ZO1-1, we found that GOPC-PDZ also contained an Ile at β2-1, which may be beneficial for accommodating a large amino acid, such as Phe or Leu, at position 4 of the internal motifs.

(c) RIMS2-PDZ: RIMS2 (Rab-3-interacting molecule 2) is a scaffold protein of the pre-synaptic active zone, which is involved in exocytosis and synaptic plasticity. It has previously been suggested that the hScrib-4, ZO1-1, and RIMS2-1 PDZ domains can recognize internal sequences due to their larger flexible carboxylate-binding loop [47]. The RIMS2-PDZ domain was found to associate with the NS5 protein of tick-borne encephalitis virus (TENV) via an internal PBM [47]. Here, we further demonstrated the internal sequence binding ability of RIMS2-1. The consensus was deduced to be [VIT]-**F**-X-[**V**T]-X-Φ, where X is any type of amino acid (Figure 3C). The internal sequences appeared to be interspersed with hydrophobic amino acids **F**, **V**, and Φ. Unlike ZO1-1, no C-terminal sequence was screened out from our system against this PDZ domain (Table 3).

**Table 3:** Number of internal and C-terminal PBMs screened from the special peptide library against 14 PDZ domains.

PDZ domains	# of internal PBMs	# of C-terminal PBMs
ZO1-1	7	5
GOPC-1	4	0
RIMS2-1	5	0
DVL2-1	14	3
LNX2-1	17	0
HtrA2-1	9	1
Syntenin1-2	12	0
Par6A-1	5	0
Whirlin-3	6	0
Harmonin-1	7	0
Dlg5-3	1	0
NHERF1-1	1	4
NHERF1-2	1	0
Par3L-1	1	0

(d) Dvl2-PDZ: Previous studies have revealed that the Dvl2-PDZ binding site is inherently flexible and can accommodate different types of ligands [18]. The C-terminal PBMs of this domain can be summarized as ΩΦGWF (Ω is an aromatic amino acid) [41], and its internal PBMs (i.e. WKDYGWIDGK, SGNEVWIDGP, etc.) were also characterized by phage display screening [18]. The preference of W in both types of ligands indicated that this aromatic amino acid may play a crucial role in recognition. Here, we identified another two internal binding patterns of Dvl2-1 (Figure 3D). The consensus of the first pattern was Φ-[**VT**I]-**L**-**Y**-[**S**G]-**S**-Φ (Figure 3D, upper panel), in which the two amino acids L and Y were conserved and two hydrophilic residues ([SG]-S) flanked by hydrophobic residues. The consensus of the second pattern was **L**-X-[**H**D]-X-X-**D**-**H**-**I** (Figure 3D, lower panel). Both patterns differed from the internal PBMs screened from the phage display library [18], further demonstrating the flexibility of the ligand binding site of the Dvl2-PDZ domain.

(e) LNX2-PDZ1: Ligand of numb protein-X2 (LNX2) protein contains four PDZ domains that might target specific substrates to mediate ubiquitin ligase activity [53]. The ligand binding specificity of the PDZ domains of LNX2 has not been studied previously. In this study, for the first time, a total of 17 unique internal PBMs were identified for the first PDZ domain of LNX2, from which a consensus was deduced as Φ-Tx-Φ-Tx-Φ (Figure 3E), which can regarded as two canonical class II C-terminal PBMs in tandem.

(f) HtrA2-PDZ: The serine protease HtrA2/Omi helps maintain mitochondrial function by handling misfolded proteins in the intermembrane space. The PDZ domain within the protein recognizes both C-terminal and internal PBMs of extended, hydrophobic tri-peptides in the phage-display system [33]. In the present study, the internal PBMs of HtrA2-PDZ were also composed mainly of hydrophobic polypeptides (Figure 3F). Though hydrophobic sequences were favored by HtrA2-PDZ in both the phage-display and yeast systems, the amino acid W might play a key role in the internal PBMs when screened in the phage-display system, whereas the residues of I, L, V, and Y were more preferred in the internal PBMs probed here. This difference may be caused by the loss of the amino acid W in our library. Nevertheless, the internal PBMs were relatively diverse, and no consensus was deduced, indicating that HtrA2-PDZ may have relatively strong plasticity, exhibiting low specificity for internal peptide recognition.

(g) Syntenin1-PDZ2: Syntenin is a multifunctional intracellular adaptor protein with two adjacent PDZ domains. The PDZ domains were not restricted to binding a unique sequence, but were capable of binding multiple peptide motifs [54]. The binding specificity of the syntenin1-2 PDZ domain was determined by the combination of three binding pockets that accommodate different motif structures via an induced-fit mechanism [55]. Here, two types of internal PBMs were detected against the PDZ domain, with preference for hydrophobic amino acids, such as A, I, V, and L. The consensus of the first group was deduced as Φ-**A-I**-X-[**A**LV]-[**I**V] (Figure 3G, upper panel), which includes two tandem canonical class II PBMs, i.e. Φ-**A-I** and **I**-X-[**A**LV]. The consensus of the second group was defined as Φ-**R**-Φ-Φ-Φ, which can be regarded as two tandem canonical class II PBMs, i.e. Φ-**R-**Φ and Φ-Φ-Φ (Figure 3G, lower panel).

(h) Par6A: Par6 is a cell polarity protein containing a single PDZ domain that has been shown to bind both C-terminal (-X**E**XLV/I-_COOH_) and internal (-X**E**XAVDX-) motifs [24], which denotes that the negatively charged amino acid **E** may be important for both recognition events. However, this amino acid was not encoded by internal fragment in our system. Nevertheless, the internal PBMs were detected for Par6A-PDZ, which contains extensively hydrophobic tri-peptides that end with the conserved aromatic amino acid F (Figure 3H). One of these internal binding sequences, #1 (-SDRGPFFP-), exhibited stronger activity in the LacZ assay than any other sequences, indicating that a strong interaction may occur between this PBM and the Par6A-PDZ domain.

(i) Whirlin PDZ3: Whirlin is a multi-PDZ scaffold protein, and defects in this protein have been shown to be involved in deafness and short cochlear hair cell stereocilia in mice and recessive deafness (DFNB31) in humans [56]. The third PDZ domain of whirlin has been suggested to interact with MPP1 via an atypical C-terminal PBM (-PQWVPVSWVY*) as well as an additional internal interaction mechanism [57]. We confirmed that this domain could bind the internal sequences with strong preference. The core of the consensus was Φ-**T**-[**F**LV]-**I** (Figure 3I), which can be regarded as two classes of tandem PBMs: Φ-**T**-[**F**LV] belongs to class II motif, whereas **T**-[**F**LV]-**I** can be viewed as a class I motif. Based on the observations that the atypical C-terminal PBM derived from MPP1, -PQWVPVSWVY*, which can be viewed as a class I motif if the last two amino acids were treated as one large hydrophobic residue, and the C-terminal PBM of this PDZ domain from Myo15A (-ITLL*), which is also a class I motif [58], we postulated that the motif -**T**-[**F**LV]-**I**- is the main interface for the internal recognition.

(j) Harmonin PDZ1: The harmonin-1 PDZ domain binds the C-terminal PBM (-LTFF*) of harmonin and an internal PBM of cadherin 23 [28]. Although this domain prefers a class I C-terminal PBM similar to that of GOPC-PDZ, it is capable of binding more diverse internal PBMs than the GOPC-PDZ domain. Two types of binding specificities have been observed for the harmonin-1 PDZ domain in this study. In the first group, a conserved aromatic amino acid (i.e., F) was located in the middle, three hydrophilic or negatively charged amino acids (e.g., S or D) on the N-terminal side of F, and four hydrophobic residues (e.g., V, I, L, and F) predominantly on the C-terminal side of F (Figure 3J, upper panel). A mimic Class I PBM (-S/T-X-F-) was present in sequences #1, #2, and #5, and another mimic Class I PBM (-SIV-) located at the extreme N-terminus of sequence #3. In the second group, two similar internal PBMs differed from group I but still contained a mimic Class I PBM (-S-A/G-C-) in the middle of these motifs (Figure 3J, lower panel), suggesting that harmonin-PDZ1 may be flexible enough to accommodate other kinds of internal PBMs.

(k) Only one internal sequence was screened out from the library when the PDZ domains NHERF1-1, NHERF1-2, DlG5-3, and Par3L-1 were used as bait sequences, respectively (Figure 3K). For the NHERF1-1 PDZ domain, four C-terminal motifs tailed with -S/T-R-L* were probed (Table 3 and Table S3).

### Characteristics of the internal binding recognition of PDZ domains

According to the intrinsic properties of the internal PBMs, they can be divided into three major categories: cryptic class I, such as those from ZO1-1, GOPC-1, harmonin-1, and whirlin-3, in which obvious motifs similar to C-terminal Class I PBMs could be found; cryptic class II, such as those from RIMS2-PDZ, HtrA2-PDZ, and syntenin1-2 in which the composition of the sequences were almost hydrophobic amino acids, or similar to those from LNX2-1 in which the hydrophobic amino acids were separated by hydrophilic residues (all of the internal PBMs may inherently mimic the canonical Class II C-terminal PBMs); cryptic class III, such as those from DVL2-1 and Par6A-1, in which diverse internal PBMs were detected.

C-terminal PBMs were probed against several PDZ domains (Table 3) due to the unexpected errors that occur during oligonucleotide synthesis or multiple DNA fragments being cloned into one vector; both situations may lead to pre-termination or elongation of the peptides. However, these unexpected results provided an opportunity for us to compare the preference or strength of recognition between the C-terminal and internal PBMs of PDZ domains. For example, the ZO1-1 domain can bind both C-terminal and internal PBMs with similar peptide numbers (Table 3 and Table S3). As internal PBMs were predominant in this special library, this result suggests that ZO1-1 may prefer to bind C-terminal PBMs. More importantly, this result also hints that uncovering the internal binding pattern for PDZ domain is difficult if a conventional cDNA or genome library with high-abundance, diverse types of unique C-terminal ligands is screened.

The proportion of hydrophobic residues in the internal PBMs was higher than that of hydrophilic ones, indicating that, for internal recognition, hydrophobic amino acids may be preferred by the binding sites of PDZ domains. In contrast, some charged amino acids (e.g., D, R, and H) are usually located at the N-terminus or C-terminus of the internal PBMs, such as those from the ZO1-1, syntenin-1, and harmonin-1 PDZ domains. The presence of these amino acids may be consistent with the Par-6-PDZ domain recognizing the internal sequence HREMAV**D**CP from the Pals1-PDZ domain, in which the negatively charged amino acid Asp forms salt bridge with the positively charged Lys residue from the carboxylate binding loop of Par-6-PDZ, and the salt bridges induces a conformational change of the carboxylate binding loop, resulting in the interaction [24]. Thus, the charged amino acids within the internal PBMs may play a similar role as Asp does in the sequence of HREMAV**D**CP.

No internal motifs were probed for some PDZ domains, which could be caused by the deficiency of five amino acids in our system as mentioned above. The Scrib-PDZ4 domain has shown an ability to bind to TBEV-NS5 via a predominant internal motif, ^219^EMYYS^223^ [27], but no positive clones were probed in our system. It might be lacking of some specific types of residues, such as E and M, which led to the failure. Based on the C-terminal binding properties of ZO1-3 and CASK-1, we tried to explain why no internal PBM was detected against these two domains from our library. The C-terminal consensus of ZO1-PDZ3 screened out from the phage system (WΩ[S/T]DWΨ-_COOH_) belongs to a Class 3a motif [41] in which the amino acid W was overwhelmingly preferred at the P^−1^ and P^−5^, indicating that the interactions with some the binding sites within the pocket of PDZ domains were highly specific. The C-terminal binding properties of the Cask-PDZ domain (WXΩFDV-_COOH_) were determined in the phage display system, in which the P^−5^ site was ready to accommodate the amino acid W. Thus, the absence of particular amino acids, such as W, may be an obstacle for probing the internal PBMs for these domains. Another reason may be that these PDZ domains may prefer longer internal PBMs, so a library with internal peptides of 10 or 12 residues may suit for screening these PDZ domains.

Although the C-terminal binding properties of two PDZ domains tend to be similar, such as ZO1-1 and HtrA2-1, or GOPC-PDZ and harmonin-1, their internal binding properties may be different in regards to several aspects, which indicates that the diversity in PDZ domain binding properties can be more easily revealed by studying their preferred internal sequences.

### Using the SP score to evaluate the internal binding ability of PDZ domains

The specificity potential (*SP*) score was used to measure the position specificities of C-terminal ligands in a previous study [41]. The *SP* score ranged from least specific (0, any amino acid is recognized) to most specific (1, only a single amino acid is recognized). The *SP* score could be an indication of the flexibility of the binding groove of the PDZ domain. The lower *SP* score suggests higher plasticity of the PDZ pocket, which accommodates more kinds of ligand residues. We introduced the *SP* score for C-terminal PBMs determined in the phage-display system [41] to assess the internal sequence binding ability of PDZ domains. From the *SP* score for human PDZ domains (Table S4), the *SP* score for P^0^ and P^−1^ of the C-terminal ligands of CASK-1 was 0.93 and 0.89, respectively; the *SP* score at the same ligand position of ZO1-3 was 0.80 and 0.98, respectively. No internal PBM was probed for these two domains in our system. In contrast, some PDZ domains that can bind internal PBMs, such as DVL2-1, ZO1-1, SNTA1-1, HtrA1-1, and HtrA2-1, had low *SP* scores (<0.70) at the P^0^ and P^−1^ positions of their C-terminal PBMs (Table S4). However, the *SP* score of P^−2^ might not correlate with the plasticity of the PDZ domain, such as the C-terminal MPBs of DVL2-1 and CASK-1, which both had *SP* scores of 0.98 at this position. Therefore, an *SP* score for the P^0^ and P^−1^ ligand position of <0.70 might be a threshold for PDZ domains binding to internal PBMs. Based on this hypothesis, some PDZ domains, such as DLG4-3, Shank3-1, MPDZ-3, PTPN13-4, and MLLT4-1 may bind internal PBMs.

## Conclusions

Here, we focused on the interaction patterns between PDZ domains and internal peptide motifs of their ligands. A systematic strategy based on Y2H screening of a special octapeptide library was developed for characterization of the internal sequences binding properties of a panel of PDZ domains. Among them, 14 PDZ domains were confirmed to bind to the internal sequences, most of them exhibited obvious consensus against one particular PDZ domain, and showed that the internal binding properties were diverse among different PDZ domains. These data suggest that the internal PBMs binding ability of PDZ domains may have been underestimated. The specificity potential (*SP*) score has potential to predict the internal PBMs binding ability of PDZ domain.

## Supporting Information

Figure S1 **Double strand cDNA template separated on 15% PAGE gel.** M: GeneRuler Ultra Low Range DNA Ladder; lane 1: 54-bp double strand cDNA template for library construction.
(TIF)

Table S1 **General information for 24 PDZ domains.**
(XLS)

Table S2 **Primers used for molecular cloning in this study.**
(XLS)

Table S3 **Detailed sequence information of the C-terminal and internal PBMs screened from the special peptide library against 14 PDZ domains.**
(XLSX)

Table S4 ***SP*** **values for C-terminal ligand subsite for some PDZ domains determined in the phage display system** [**41**] **.**
(XLS)
