# Supplementary figures and images for: Characterization of Diverse Internal Binding Specificities of PDZ Domains by Yeast Two-Hybrid Screening of a Special Peptide Library
Source: PLoS One. 2014 Feb 4;9(2):e88286. doi: 10.1371/journal.pone.0088286 (PMC3913781; doi:10.1371/journal.pone.0088286)

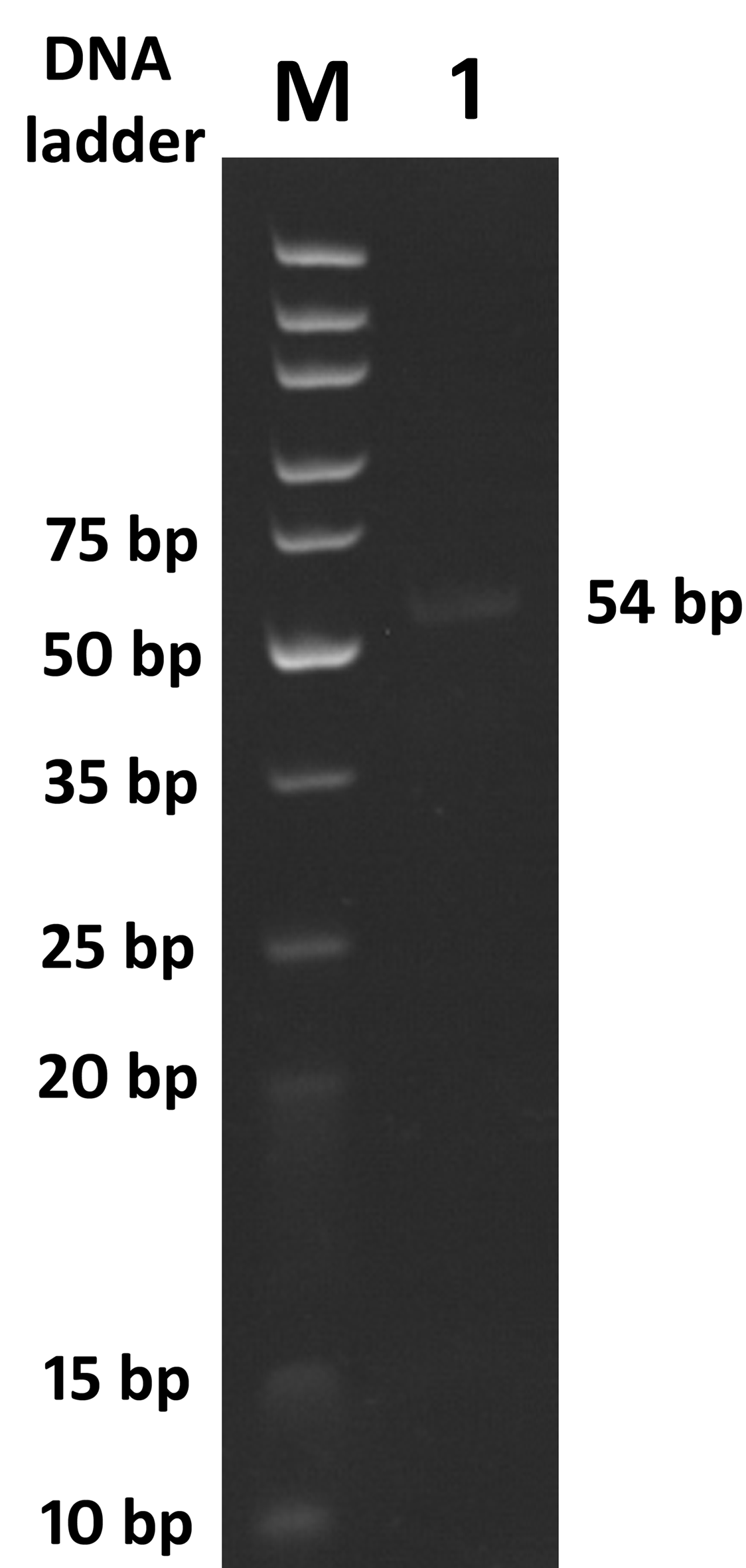

Supplement: Figure S1 — Double strand cDNA template separated on 15% PAGE gel. M: GeneRuler Ultra Low Range DNA Ladder; lane 1: 54-bp double strand cDNA template for library construction. (TIF) [file pone.0088286.s001.tif]
